# Research Tools for the Measurement of Pain and Nociception

**DOI:** 10.3390/ani6110071

**Published:** 2016-11-11

**Authors:** Craig Johnson

**Affiliations:** Animal Welfare Science and Bioethics Centre, Institute of Veterinary, Animal and Biomedical Sciences, Massey University, Palmerston North 4474, New Zealand; c.b.johnson@massey.ac.nz; Tel.: +64-6-356-9099

**Keywords:** pain measurement, research techniques

## Abstract

**Simple Summary:**

Pain is an integral aspect of many diseases and it is important to be able to measure it in the clinic so that the progression of disease and the animal’s response to treatment can be monitored. When research into pain is undertaken, it is also important to be able to measure the pain, but this time the aim is to provide meaningful results that will further our understanding of the mechanisms of pain or how it can be better treated. This change in emphasis between clinical and research measurement of pain means that the advantages and disadvantages of the many ways in which pain can be measured influence the choice of the most suitable technique and the way in which it is used. It is important to carefully select the most appropriate methodologies so that the data generated are relevant to the hypotheses being tested.

**Abstract:**

There are many ways in which pain in animals can be measured and these are based on a variety of phenomena that are related to either the perception of pain or alterations in physical or behavioural features of the animal that are caused by that pain. The features of pain that are most useful for assessment in clinical environments are not always the best to use in a research environment. This is because the aims and objectives of the two settings are different and so whilst particular techniques will have the same advantages and disadvantages in clinical and research environments, these considerations may become more or less of a drawback when moving from one environment to the other. For example, a simple descriptive pain scale has a number of advantages and disadvantages. In a clinical setting the advantages are very useful and the disadvantages are less relevant, but in a research environment the advantages are less important and the disadvantages can become more problematic. This paper will focus on pain in the research environment and after a brief revision of the pathophysiological systems involved will attempt to outline the major advantages and disadvantages of the more commonly used measurement techniques that have been used for studies in the area of pain perception and analgesia. This paper is expanded from a conference proceedings paper presented at the International Veterinary Emergency and Critical Care Conference in San Diego, USA.

## 1. Pain Pathways and the Importance of Cognition

The last two decades have seen great advances in our understanding of pain [1]. In particular, the molecular biology of pain and the way in which the central nervous system perceives and processes pain have been transformed. Molecular techniques have revealed previously unknown nociceptive mechanisms at the cellular level [2], and functional imaging techniques have demonstrated the importance of the cerebral cortex in the perception of pain and, in particular, the role of the rostral cingulate gyrus in this process [3]. This paper is expanded from a conference proceedings paper presented at the International Veterinary Emergency and Critical Care Conference in San Diego, CA, USA [4].

A detailed discussion of central pain pathways is beyond the scope of the present paper, but three aspects will be discussed further because they are of central importance when selecting techniques to measure pain in a given circumstance and also in interpreting the results of these techniques. For further details on central nociceptive pathways generally, see [5]. The three areas of direct relevance are:

- The link between pain perception and cognitive function.
- The role of the rostral cingulate gyrus in pain perception.
- Distinction between the perception of pain and the expression of pain-related behaviour.

The link between pain perception and cognitive function is important as this concept is central to understanding the kind of information that can be gained from measuring pain and nociception in different ways. In general, different measures of nociception/pain will reflect information either about the function of the entire pathway or will be limited to a specific part of it. The extent to which different measures reflect activity in different parts of the system is a very important consideration when choosing a technique to measure pain for a given research application. It should be remembered that the parts of the system do not operate in isolation and even though there may be no involvement of a particular part of the pain pathway in the generation of a particular result, nonetheless, that part of the pathway may be influencing the way that the system as a whole is functioning. For example, noxious stimulation can result in changes in the cardiovascular system such as tachycardia and hypertension. This reflex response does not directly involve the cerebral cortex, but alterations in cortical function can influence these responses via descending pathways that project to the areas that are directly involved [6].

The current view of the rostral cingulate gyrus is that it receives input from the nociceptive pathways and from other areas of the central nervous system including systems involved with emotion and arousal and integrates this information to make a decision about the extent to which a particular stimulus will be perceived as painful [7]. In this sense pain perception is considered to be a decision about a particular stimulus and the rostral cingulate gyrus represents the point in the pathway at which this decision is made. This is where nociception becomes pain. An animal’s state of cognitive function is therefore of critical importance to the extent to which it will perceive and suffer pain as the result of a particular noxious stimulus [8]. An understanding of this process is essential when utilizing different ways of measuring pain.

There are fundamental differences between the perception of pain and the efferent effects (behaviour, hormonal responses, etc.) of that pain. Pain perception is internal and subjective and may not correlate well with external and objective signs of pain as assessed by, for example, behaviour [9]. In a physiological sense, pain perception and behaviour are very different phenomena with different underlying causes that can alter them in different ways. Pain perception is strongly influenced by the history and mental state of an individual and especially by its history of previous pain perception, its degree of anxiety and the extent to which it feels in control of its situation [6]. Pain-related behaviour is strongly influenced by an individual’s cultural environment and its relationships with other animals and people [10]. Pain-related behaviours communicate information about pain and are learned from peers when animals are young [11]. The extent to which an individual from a particular cultural background will display pain-related behaviour will change depending on how this information is likely to be used in that animal’s cultural context. An obvious example of this is the difference in the tendency of herbivores that live in large herds to demonstrate pain-related behaviour compared to that of carnivores that hunt in groups. Overt demonstration of pain-related behaviour may increase the likelihood that an individual herbivore becomes a target of predation. In these species, pain-related behaviour tends to be very subtle, for example in sheep the position of the ears relative to the head [12]. In hunting carnivores, predation by other animals is less likely and so pain-related behaviour tends to be more overt and easier to detect.

## 2. Options for the Measurement of Pain

When considering techniques to measure pain in research environments, care must be taken to ensure that those selected provide robust information that can be used to address the objectives of the study. Techniques will have characteristic features that may be advantageous or disadvantageous in different situations, but they will also provide different kinds of information that will be more or less relevant to the study.

The electrophysiology of peripheral sensory nerves, for example, is not usually considered to be a good technique to evaluate pain because of the many factors that can alter pain perception that occur after the nociceptive action potentials have passed through the sensory nerve. This technique was, however, used to excellent effect [13] to investigate the time course of the effects of scrotal ischaemia induced by rubber ring castration in lambs. In this study, effects due to causes other than ischaemia were removed from the collected data by recording the electrical activity of the sensory nerves innervating the scrotum. The spontaneous activity of these nerves and their response when the scrotum was mechanically stimulated were used to demonstrate the functional effects of the application of the rubber ring. These measurements were not influenced by effects occurring further along the nociceptive pathway and so were easily attributable to the development of ischaemia in the tissues of the scrotum.

**Figure 1:**
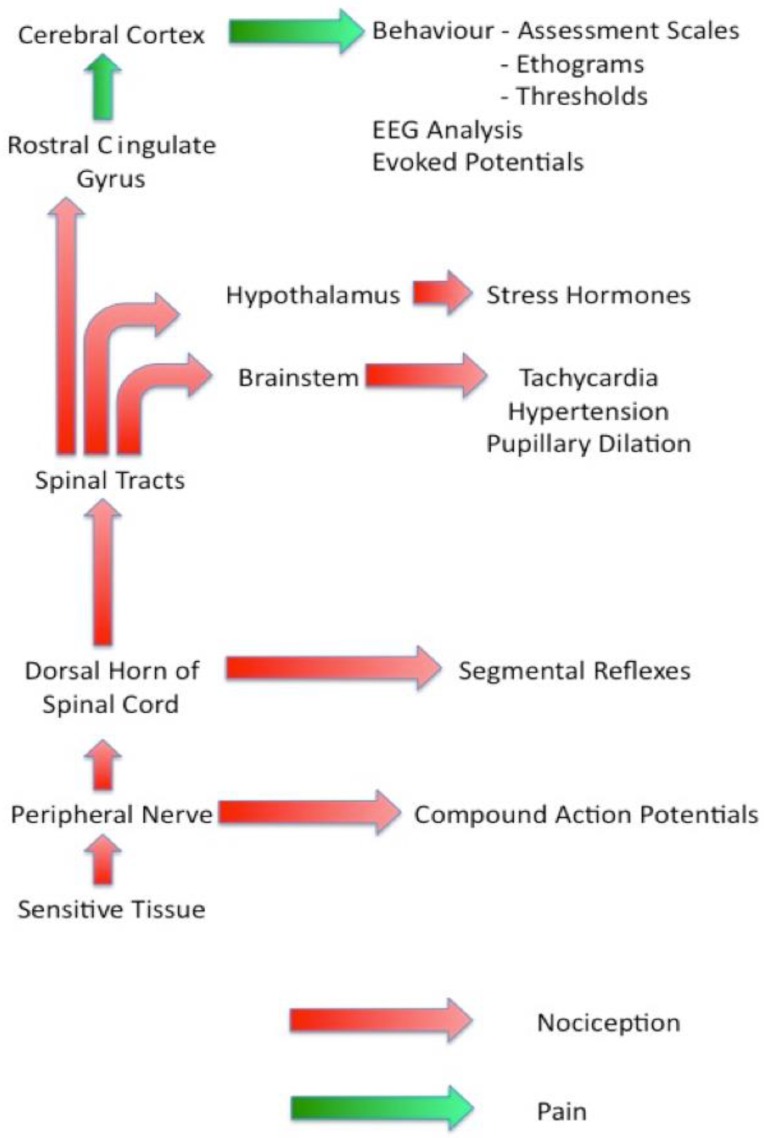
Diagrammatic representation of the sensory nociceptive pathways. EEG: electrocardiograma.

Figure 1 is a diagrammatic representation of the sensory nociceptive pathways with each of the groups of assessment techniques identified by the point at which they diverge from the main pathway. This information should be used in conjunction with the features of each group of techniques discussed below when selecting suitable methodologies to be used in a particular study. Note the change in terminology from ‘nociception’ to ‘pain’ that occurs as the signals pass through the rostral cingulate gyrus.

## 3. Features of Techniques

This section will consider a number of commonly used pain assessment techniques and outline the features that influence their suitability in different situations in the research environment.

### 3.1. Physiology-Based Techniques

These techniques rely on the physiological effects of noxious stimulation, usually, but not always through the actions of the autonomic nervous system. A variety of methodologies work in this way including heart rate and measures of heart rate variability such as RR interval analysis [14], blood pressure [15], pupillary dilation [16] and probably infra-red thermography [17]. In general, they are easy to use and generate objective data that can be recorded over the short, medium and long terms. This means that these techniques can record responses to noxious stimulation by the second, by the minute or even by the hour. The ease of data collection and the objective nature of the data make them easy to work with and facilitate statistical analysis, but there are a number of theoretical and practical drawbacks.

Theoretically speaking, the reflexes which control these autonomic responses to noxious stimulation are modulated in the cardio-respiratory centres of the medulla oblongata or motor centres in the brain stem such as the parasympathetic nucleus of the third cranial nerve (Edinger–Westphal Nucleus). They do not necessarily reach to the cognitive centres and so are not necessarily evidence of pain perception. This modulation below the level of the higher centres may be why they correlate poorly with pain perception in clinical studies [18].

The main practical drawback of these techniques is that they are not specific to noxious stimulation and so should be interpreted with care in studies where changes in these variables due to other causes are not controlled. For example, increases in heart rate could be due to exercise, anxiety, hypovolaemia, etc. and controls should be in place to prevent these from being erroneously interpreted as pain.

Physiology-based techniques are seldom used alone as indicators of pain, but they can be useful when combined with other methodologies.

### 3.2. Pain Thresholds

Techniques relying on an animal’s first response to a progressively increasing stimulus are referred to as pain threshold tests or quantitative sensory testing. These have proved very useful in a variety of settings [19,20,21], but as with all techniques, care has to be taken to ensure that a valid result is obtained. When using these techniques there are a number of factors that must be considered.

Responses to stimuli have both reflex and cognitive components. Reflex components are processed in the spinal cord within the dermatome of the stimulus (segmental reflexes) and also in association with other dermatomes (long reflexes). They usually involve withdrawal of the stimulated limb or twitching of the musculature in the stimulated area. They are usually limited to the stimulated dermatome, but sometimes also involve long spinal reflexes with responses in other segments of the body. An example of such a long reflex is the cutaneous trunci (panniculus) reflex. Cognitive components of a response are those that include the higher centres of the central nervous system and are indicated by more complex behaviours that indicate cognitive perception of and aversion to the stimulus [22]. These responses are usually more global than the localised reflex responses. When performing threshold testing, it is important to be certain that the measured response is cognitive rather than solely reflex in nature.

Practically speaking it is important to carefully control the research environment to ensure that the noxious stimulus is applied under the same circumstances at each test. Factors such as ambient temperature, environmental noise, unfamiliar people, etc. can all have effects on measured thresholds and lead to inaccuracies. Care should also be taken to prevent animals from anticipating the application of a stimulus by association with the routine that leads up to its application. Even low levels of pain are strong motivators to learning and animals can quickly come to associate very subtle visual or auditory stimuli with the next application of a noxious stimulus. This can lead them to display altered responses that are not solely due to altered pain thresholds [21].

Habituation and sensitisation are also potential issues with protocols that measure pain thresholds. These phenomena cause responses to noxious stimuli to decrease (habituation) or increase (sensitisation) over time. These changes can be caused by low-grade tissue damage at the site of stimulation, the development of conditioned reflexes due to the stimulus or other sensory cues presented at the same time and a number of other extraneous factors. In a study using mechanical thresholds to measure the effect of a number of analgesic drugs on sheep [23], it became apparent that animals quickly began to associate the sight of the researcher’s hand approaching the stimulus actuator and to respond to this rather than to the noxious stimulus itself. The actuator had to be placed out of the animal’s field of view in order to be able to record genuine responses to the noxious stimulus itself [24].

Care should be taken to limit noxious stimuli to below levels that lead to even very slight inflammation [21]. When a new stimulus modality is being used for the first time in a particular circumstance or on a particular experimental population, a pilot study to document the stability of responses over time is a very useful way to be sure that the data collected relate to genuine changes in pain thresholds. Subtle changes in the protocol such as site of stimulation, diameter of probe or posture of the animal during testing can result in altered results [25]

Several different kinds of stimuli including mechanical, thermal and electrical can be used to test pain thresholds. Each of these has particular advantages and disadvantages (Table 1) and care should be taken to use each of them appropriately.

Mechanical stimulators stimulate mechanical transducers and so closely mimic many of the noxious stimuli encountered outside of the laboratory. They are easy to control and the stimulus can be terminated very quickly. They are also stable over time and produce little tissue damage. Their disadvantages result from the complexity of the way in which mechanoreceptors respond to the application of force. The effects of changes in the size and shape of the probes used in different devices are not easy to model and this means that it is difficult to compare the results of studies that use different equipment [26]. There is also the possibility that co-stimulation of other senses such as touch could be responsible for the recorded responses.

Thermal stimulators do not cause co-stimulation of receptors other than thermoceptors and the characteristics of the probe are much less important than with mechanical stimulators making comparison of studies performed with different devices easier. Control and termination of the stimulus is more difficult and over time this can cause problems with tissue damage leading to sensitisation. Environmental temperature also has an influence on results obtained with thermal stimulators and this must be accounted for by the experimental methodology [21].

Electrical stimulation is very easy to control and when used properly causes no tissue damage. The major problem with this method of stimulation is that it bypasses transducers in the tissue and stimulates all sensory nerves directly. This produces a mixed sensation that is not entirely noxious in character and is quite different from noxious stimuli encountered outside the laboratory.

### 3.3. Hormonal Assays

A variety of hormones have been used in studies of pain perception. These are usually linked to the ‘stress’ response of the hypophyseal–pituitary axis and exploit the actions of noxious stimuli as stressors [27]. The most common hormones measured in studies of this nature are the glucocorticoid hormones (cortisol and corticosterone—depending on the species studied), but other hormones such as beta-endorphin, oxytocin and adrenaline have also been used [28,29,30]. Cortisol and corticosterone are convenient hormones for this kind of study because they appear in measurable concentrations in plasma, saliva, urine and other body fluids, are robust molecules that are easy to keep stable in collected samples and are fairly easy to measure. This makes studies possible in a variety of situations in the field where there can be considerable delay between sample collection and analysis [31].

When using stress hormones as part of pain-related studies, a number of factors must be considered. The stress response itself is a generalised response to environmental stressors that include: pain, hypovolaemia, hypoxia, exercise, capture, overcrowding, boredom, poor nutrition, etc. Any study using stress hormones to investigate pain must ensure that all other stressors are controlled so that changes in the hormones measured can be reliably interpreted as being due to the noxious stimulus used in the study.

The magnitude of the response must also be carefully considered. A noxious stimulus will only result in a measurable stress response if it is of a sufficient magnitude and duration. Very minor noxious stimuli or those applied for very short durations will not produce an increase in stress hormones above their basal levels. In addition, there is a maximal possible increase in these hormones (a maximal stress response), such that noxious stimuli that produce maximal responses cannot be distinguished from each other in terms of stress response alone. For example, studies that investigate different techniques of analgesia for surgical procedures in wild animals may not be able to distinguish between groups by magnitude of stress response if the (non-painful, but very stressful) act of capturing the animals in the study provokes a maximal stress response [32].

### 3.4. Behavioural Analysis

In clinical situations, behavioural techniques used to assess pain usually follow the approach of a carer scoring pain on some kind of scale. Examples of these are simple descriptive pain scales [33], visual analogue scales [34] and the more involved composite pain scales [35]. These are all clinically very useful, but have a number of drawbacks (Table 2) that make them less suitable in research situations where the primary aim is to derive statistically meaningful data. In particular, the non-parametric and categorical nature of simple descriptive pain scores can make appropriate analysis of data difficult and can lead to significant reductions in the statistical power of studies.

Conversely, techniques based on ethograms are usually too time-consuming and cumbersome to use in clinical environments, but the statistical utility of such techniques make them extremely powerful in a research setting. In particular, the ability of such techniques to identify and validate novel pain-related behaviours has led to significant advances in the clinical identification of pain in species that show few or only subtle pain-related behaviours. Notable examples of this include pain-related behaviour in horses [36], identification of arch and writhe behaviours in rats [37] and mouse grimace scale [38].

### 3.5. Electrophysiology

Electrophysiological techniques measure electrical activity at some level of the nervous system that is related to the noxious stimulus or the perception of pain arising from it. The advantages and disadvantages of each technique in this category depend upon the nature of the response at the site of measurement and also upon the degree of restraint or control of the animal required to enable the data to be recorded. For example, auditory evoked responses can be recorded from unsedated animals, but somatosensory evoked potentials utilise a stimulus that is itself painful and so require profound sedation or general anaesthesia. The use of electrophysiological techniques to assess pain in animals has been reviewed [39].

Electrophysiological techniques commonly used in studies of nociception and pain include many different techniques such as recording from peripheral nerves [13], analysis of the electroencephalogram (EEG) [40], recording of evoked potentials, usually the somatosensory-evoked potential (SEP) [41]. Electrophysiological techniques can be used in many ways, and a full consideration of them is beyond the scope of this review. An example of the use of each of these techniques will be considered here in order to outline some of the advantages and disadvantages.

Recording from peripheral nerves [13] has been considered above.

Recordings of EEG made from anaesthetised lambs have been used to investigate the effect of age on the perception of pain resulting from castration by rubber ring [40]. The development and use of the minimal anaesthesia technique used in this study have been reviewed elsewhere [42]. This technique can indicate the degree of pain due to a noxious stimulus using variables derived from the EEG that are robust to minimal anaesthesia. Although this methodology is relatively complex and requires very precisely controlled general anaesthesia, it is statistically powerful because of the high mean change to standard deviation ratio and the objective nature of the data generated. This means that it can produce results using smaller group sizes than many other methodologies. In addition, because the animals are anaesthetised throughout the period of data collection, routine analgesia can be provided prior to recovery. This reduced group size and low ethical cost to all animals including those in control groups makes this technique very attractive as a way to minimize the ethical cost of pain research in animals.

A study utilizing evoked potentials derived from the EEG [41], investigated the degree to which the magnitude of a noxious stimulus and its perceived unpleasantness are linked. The methodology of evoked potentials and their use in pain assessment in animals has been reviewed elsewhere [39]. Evoked potentials have the advantage that they can isolate the electrical activity of a particular neurological pathway from the background of the EEG and so provide information about the likely anatomical location of responses to noxious stimulation. Their derivation relies on analysis of the EEG following repeated stimulation; often a large number of repeats are needed. This results in the key disadvantages that the experimental subjects need to be in a similar neurological state during the delivery of all stimuli and that great care needs to be taken to ensure that each stimulus in a train does not have any cumulative effect on the response to subsequent stimuli.

### 3.6. Molecular Biology

Molecular biological techniques are beginning to find a place in the study of pain. Such studies usually focus on ultrastructural aspects of the pain pathways or changes to these pathways seen in conditions that are chronically painful. These techniques are currently transferring to the study of acute pain and are demonstrating, for example, mechanisms of hyperalgesia by examining changes in protein expression in the dorsal horn following surgical stimulation [43]. Recently, preoperative meloxicam has been shown to influence the expression of mRNA in plasma for four different cytokines, interleukin (IL)-8, prostaglandine (Pg)E synthase, IL-1B and IL-1A in calves undergoing disbudding by thermocautery (unpublished data). Studies such as these may result in the development of biochemical markers for nociception and possibly even pain.

## 4. The Power of Combinations

Combination of different techniques of pain assessment can be used to enhance the information gained from an experimental study and to offset some of the disadvantages of the techniques chosen. Where techniques are combined, care needs to be taken to ensure that the overall methodology does not compromise any of the data collected and that the techniques chosen are complimentary in the kind of information that they will provide. Great care should be taken to decide on the kind of information that is required and to select techniques that deliver this information in a manner as succinct and robust as possible.

Much veterinary research aims to consider the effects of noxious stimulation in clinical and animal husbandry environments rather than examining particular pathophysiological processes associated with pain perception. For example, a researcher may be interested in developing an optimal technique of analgesia for a particular surgical procedure such as fixation of a ruptured cranial cruciate ligament in the dog. The progression from initial injury, together with the many underlying predisposing factors, through diagnosis, options for surgical treatment, acute pain management, progression to longer term analgesia, possible complications and eventual resolution constitutes a very complex clinical scenario and it is naïve to assume that a single study of simple experimental design will be able to adequately account for all of these factors. In such situations it is much more powerful to dissect out particular aspects of the clinical scenario, to design experimental studies that address these in isolation and then to build an understanding of the overall situation onto which particular clinical interventions can be superimposed to evaluate their effects. Different aspects of the clinical scenario may be suited to different research approaches and so studies that isolate each of these may utilize different methodologies that are then combined to form a complete picture.

This approach has resulted in very complete understanding of a number of clinical procedures. Castration in lambs is a good example. A large number of research techniques including examples from every category discussed in this review have contributed to our understanding of the pain of castration in lambs with more than 120 relevant papers in the literature. This understanding is now forming the basis of studies investigating ways in which analgesia for castration can be provided to lambs in a way that is practically and economically viable in the commercial sector.

## 5. Conclusions

Techniques suited to the measurement of pain are subtly different in the clinical and research environments. This is due to the different uses to which information about pain is put, in these two environments. Despite the subtlety of the differences between these environments, they often require very different approaches to data collection. The aims and objectives of studies investigating pain must be clearly set out and great care taken to use research methodologies and experimental designs that can appropriately address these.

## Figures and Tables

**Table 1:** Advantages and disadvantages of commonly used threshold testing modalities.

Modality of Stimulus	Advantages	Disadvantages
Mechanical	Stimulation via mechanical transducers	No standard for size and shape of probe
	Easy to control and terminate stimulus	Difficult to compare between studies with different equipment
	Stable over time	Co-stimulation can be a problem
	Little tissue damage	
Thermal	No co-stimulation	Control and termination more complex
	Probe characteristics less important	Tissue damage may be an issue
	Easy to compare different studies	Background temperature is a variable
Electrical	Little tissue damage	Bypasses transducer
	Very easy to control and terminate stimulus	Texture of sensation mixed

**Table 2:** Advantages and disadvantages of simple descriptive pain scales.

Advantages	Disadvantages
Rapid to administer	Data are categorical (non-parametric)
Little extra paperwork	Variations between assessors
Conceptually simple	Subjective measurement
Non-invasive	
